# Adjusting the *15-method* to Danish general practice: identification of barriers, facilitators, and user needs

**DOI:** 10.1186/s12875-024-02508-z

**Published:** 2024-07-06

**Authors:** Peter Næsborg Schøler, Jens Søndergaard, Sanne Rasmussen, Anette Søgaard Nielsen

**Affiliations:** 1 Unit for Clinical Alcohol Research, Research Unit of Psychiatry, Department of Clinical Research, University of Southern Denmark, Odense, Denmark; 2 Research Unit of General Practice Odense and Esbjerg, Department of Public Health, University of Southern Denmark, Odense, Denmark; 3 BRIDGE, Brain Research - Inter Disciplinary Guided Excellence, University of Southern Denmark, Odense, Denmark; 4 Department of Psychiatry Odense, Mental Health Services Region of Southern Denmark, Odense, Denmark

**Keywords:** Alcohol use disorder, Primary healthcare, Screening and brief intervention, Participatory research, Physician-patient relations, Implementation science

## Abstract

**Background:**

The *15-method* is an opportunistic screening and brief intervention tool for alcohol-related problems in primary healthcare. A Danish feasibility study of the *15-method* indicated that adjustments were needed to improve its contextual fit to Danish general practice. This adjustment process was conducted in two parts. The first part focused on identifying barriers, facilitators, and user needs for addressing alcohol using the *15-method*. The second part will address the identified barriers and user needs to finalize a Danish version of the method. This study reports on part one of the adjustment process.

**Methods:**

Semi-structured individual interviews and focus group interviews with healthcare professionals (*n* = 8) and patients (*n* = 5) from general practice in Denmark. Data analysis was conducted using thematic content analysis. The results were condensed into two focus areas that will form the basis for user workshops in part two of the adjustment process.

**Results:**

The main barriers for addressing alcohol using the *15-method* were patients and healthcare professionals not having the same agenda, having difficulty opening a conversation on alcohol, and workflow in the practices. Main facilitators included high interpersonal skills, taking the patient’s perspective, and good routines and interdisciplinary work. Suggested adjustments and additions to the method included digitalization, visual icebreakers, quotes and examples, and development of a quick guide. The identified focus areas for user workshops were Communication and Material, and Integration to Workflows.

**Conclusion:**

Healthcare professionals found the opportunistic screening approach exemplified by the *15-method* to be beneficial in identifying and addressing alcohol-related problems. They appreciate the method’s structured framework that assists in presenting treatment options. Identified adjustment areas to the *15-method* will lay the groundwork for future efforts to develop a finalized Danish version of the *15-method*.

**Supplementary Information:**

The online version contains supplementary material available at 10.1186/s12875-024-02508-z.

## Introduction

The *15-method* is an opportunistic screening and brief intervention (SBI) tool for alcohol-related problems in primary care [1]. The present study is part of a series of studies on the feasibility testing, adjustment, and evaluation of the *15-method* in Danish general practice.

The general practitioner (GP) is in contact with a large part of the population and is often regarded as a trusted advisor [2]. This makes general practice an opportune place to identify alcohol-related problems [3, 4]. Danish GPs have the highest number of face-to-face consultations among Scandinavian GPs, with a yearly average of almost eight contacts per patient listed [5]. Despite this, SBI tools for alcohol have a low degree of implementation and effectiveness [6–8]. The implementation challenges of alcohol interventions in healthcare often relate to stigma [9, 10], time constraints [11], policy making [12, 13], and insufficient focus on the context [8, 14].

The *15-method* intertwines elements from alcohol treatment and from SBI research into a structured, stepped-care framework [15]. The name “*15-method*” serves a dual purpose: it targets patients who score over 15 points on the Alcohol Use Disorder Identification Test (AUDIT) [16], indicating a significant concern with alcohol use, and it denotes the consultation duration of 15 min per consultation. The method is designed into three progressive steps that can be integrated into the patient’s existing consultation schedule for efficiency and effectiveness.

The first of the three steps capitalizes on already scheduled consultations to conduct opportunistic screenings. Thus, it does not require a separate appointment but instead utilizes interactions such as routine examinations or discussions about symptoms or lab results to assess potential alcohol-related issues. A brief evaluation of the patient’s drinking habits is paired with concise advice and directions on how to access further resources, including completing the AUDIT in preparation for the patient’s next visit.

The second step is a deeper assessment of the patient’s symptoms in the context of their alcohol use, incorporating a health check when necessary [17]. Healthcare professionals (HCPs) pay particular attention to AUDIT scores, lab results, and any patient-specific information such as sleep patterns and blood pressure. Patients then receive personalized feedback and are informed about the next step, which can range from a simple follow-up to referral for specialized treatment or involve progression to the third step of the method.

The third and final step features up to three consultations centered around cognitive-behavioral therapy and motivational interviewing principles to guide patients towards self-driven change [18, 19]. Each session focuses on a specific theme and is complemented by practical homework assignments tailored to foster skills such as self-monitoring, recognizing risky scenarios, and finding drinking alternatives. Goals and the intensity of the treatment are collaboratively determined through shared decision-making [20], potentially incorporating pharmacological aids like disulfiram, acamprosate, nalmefene, and naltrexone in line with national guidelines.

Continuous monitoring of blood results, AUDIT scores, and other health indicators throughout the three steps serves not only to track progress but also to strengthen patient motivation and ensure a supportive, informed, and tailored treatment.

Finn et al. found the *15-method* to be a promising approach for treating alcohol problems in Swedish general practice [1, 21] and the *15-method* is currently being implemented in a mental health setting in the region of Stockholm, Sweden. In a Danish feasibility study of the *15-method* [22], GPs, nurses, and patients found the *15-method* acceptable and feasible to use in a primary care setting. The Danish feasibility study also showed that adjustments to the method’s content and form prior to large-scale evaluation could improve its fit and potentially its effectiveness. To make these adjustments, we engaged in a two-part participatory design process [23]. Part one focused on learning more about the patients’ and healthcare professionals’ views on the adjustments needed for use in Denmark. Part two will address the identified adjustment areas in user workshops to finalize the Danish version of the *15-method*.

The present study reports on the first part of the adjustment process aimed at identifying barriers, facilitators, and user needs for addressing alcohol using the *15-method* in a general practice setting. Specifically, we investigated (i) what happened in the communication about alcohol in relation to the *15-method* including how its structure and material affected potential barriers to addressing alcohol, and (ii) which aspects of the *15-method* the patients and healthcare professionals found facilitated a discussion about alcohol and which adjustments they considered most important for a better contextual fit to Danish general practice.

## Methods and material

### Study design

The present study was a qualitative interview study with healthcare professionals and patients from Danish general practice. The reporting follows the Consolidated Criteria for Reporting Qualitative Research (COREQ) guidelines [24]. The overall adjustment process of the *15-method* to Danish general practice follows the Medical Research Council’s guideline on developing and adjusting complex interventions [25].

### Setting

Danish general practice acts both as a first-line provider and a gatekeeper between the primary and secondary healthcare sectors and is for most patients the first point of contact with the healthcare system [26]. Denmark has approximately 1650 practices with 3500 GPs [27], and 99% of Danish residents are affiliated with a GP [28, 29]. Each GP has 1500–2000 listed patients. GPs employ their own staff and are self-employed on contracts that detail their opening hours and required education and services, as well as the reimbursements through the tax-funded healthcare system that make consultations free of charge for patients [26]. Practices vary in size from solo practitioners to company practices with five or more GPs in a single unit, and GPs have a high degree of freedom to schedule their workflow in the practice, e.g. consultation times and administrative work. Practices engage in continuous quality improvement activities and self-monitoring, for example through scheduled staff meetings, quarterly and annual plans, and required continued education on topics selected by the Danish Organization of General Practitioners and the Danish Regions (administrative entity between the municipalities and the government).

### The research team

The research team consisted of Peter N. Schøler (PNS), Anette S. Nielsen (ASN), Jens Søndergaard (JS), and Sanne Rasmussen (SR). PNS is a medical doctor and PhD student at the Unit for Clinical Alcohol Research, University of Southern Denmark (SDU). ASN is head of the Unit for Clinical Alcohol Research and professor in clinical alcohol research. JS is a general practitioner and professor and head of the SDU Research Units for General Practice in Odense and Esbjerg. SR is a general practitioner and associate professor at the Research Unit for General Practice Odense.

### Characteristics and preconceptions

PNS conducted all interviews in the present study. PNS has research experience within psychiatry and general practice through his positions as research assistant and PhD student, and clinical experience from general practice and somatic hospitals in Denmark. PNS has interviewing experience through his medical training, research, and qualitative research network groups. PNS has knowledge of the *15-method* through clinical work and his research.

PNS, ASN, and JS collaborated on the earlier feasibility study of the *15-method* in Danish primary care and continue to collaborate on related projects. Their common research interest is to investigate, develop, and evaluate practical and evidence-based approaches for treatment of alcohol disorders.

### Recruitment

We aimed to gather knowledge from healthcare professionals, here referring to GPs and nurses, with and without experience of using the *15-method*. HCPs with experience of the *15-method* would be able to identify specific details to adjust, while those without prior experience would be able to help generate new ideas or insights as to what a “perfect” brief intervention for alcohol problems could look like in primary care. We aimed to include both GPs and nurses as their roles using the method differ [22]. We also aimed to include HCPs from both urban and rural practices to capture differences in workflow and patient characteristics. We invited GPs and nurses working in general practices in the Region of Southern Denmark via email. PNS visited one practice facility in person as a follow-up to the email invitation.

We recruited HCPs in a criteria-based purposive sampling approach [30]. The sampling criteria were: (1) HCPs with and without experience in the *15-method*, (2) HCPs from both rural and urban practices, and (3) HCPs from both solo and partnership practices.

Patient recruitment mirrored the strategy used for HCPs, aiming to include individuals with varying levels of experience with alcohol-related issues. We sought to include patients both with and without prior or ongoing alcohol problems to identify necessary adjustments to the *15-method* across different levels of alcohol-related issues. Given that the method targets a broad patient population—from hazardous use to moderate dependence, as well as those with potential risky alcohol use but no current problems—we aimed to recruit patients with diverse experiences with alcohol.

Participating patients were recruited in a snowball sampling approach [30]. Contact was established through the research teams professional networks and through a user panel affiliated to the Danish non-profit, government supported interest organization Alcohol & Society, who works towards reducing harmful alcohol consumption in Denmark [31]. The user panel consists of persons who wish to share their experiences with alcohol-related problems for the benefit of research, treatment, and public information. Patients were invited via email.

All participants received information on the study design and purpose via e-mail and were offered a follow-up phone call to answer any questions related to the study. HCPs were informed that interviews would be conducted as focus groups either online or in person at their practice facility. Patients were informed that they could choose to participate in either an online focus group or an individual interview conducted online or in their own home.

We invited healthcare professionals from the five practices that participated in the feasibility study of the 15-method in Denmark, as well as from one additional practice facility. A total of five GPs and three nurses from three different practices agreed to participate. Among them, three GPs and one nurse had experience working with the *15-method*. The HCPs who declined the invitation stated lack of time and low staff resources as the main reasons for not participating.

Five patients were invited, and all agreed to participate. Two patients were recruited through the user panel, and three via the snowball sampling initiated in the research group’s network.

Table 1 presents participant characteristics.

**Table 1:** Characteristics of the eight healthcare professionals and five patients who participated in the study interviews

Participant no	Occupation	Gender	Practice no	Interview	Participated in the Danish feasibility study of the 15-method
HCP 1	Nurse	Female	3	Focus group 2 (in person)	No
HCP 2	Nurse	Female	3	Focus group 2 (in person)	No
HCP 3	General practitioner	Male	3	Focus group 2 (in person)	No
HCP 4	General practitioner	Female	3	Focus group 2 (in person)	No
HCP 5	General practitioner	Male	1	Focus group 1 (video)	Yes
HCP 6	Nurse	Female	1	Focus group 1 (video)	Yes
HCP 7	General practitioner	Female	2	Focus group 1 (video)	Yes
HCP 8	General practitioner	Female	2	Focus group 1 (video)	Yes
Patient 1	-	Male	-	Individual (in person)	No
Patient 2	-	Female	-	Focus group 3 (video)	No
Patient 3	-	Male	-	Focus group 3 (video)	No
Patient 4	-	Male	-	Focus group 3 (video)	No
Patient 5	-	Male	-	Focus group 3 (video)	No

### Description of participants and relations prior to study commencement

Three out of the five patients had experience with alcohol-related problems. These problems varied from mild to severe and included ongoing concerns about current alcohol-related issues. The patients drew from their personal experiences related to e.g. primary care, alcohol, and lifestyle change. The patients where all native Danes and had relevant experience in the Danish healthcare system. The patients were not affiliated to the participating practices.

HCPs from two of the practices had participated in the feasibility study of the *15-method* in Danish primary care and were thus familiar with the research team. The HCPs from the third practice, unfamiliar with the method, knew of PNS through his clinical work unrelated to his research. One patient knew of ASN through personal networks.

### Data collection

PNS collected all data. Data were collected through interviews to provide the level of detail needed to answer our research questions. The interviews had a duration of 45–60 min and were recorded as audio files without field notes or repeat interviews. Transcripts were not returned.

#### Patient interviews

One patient preferred to participate in an individual interview rather than a focus group and was interviewed in the patient’s own home. The other four patients preferred the focus group format and were interviewed in a group session via video. They were alone in their home during the interview and participated via their own computers.

#### Healthcare professional interviews

The HCP interviews consisted of two focus groups. In the first group, all HCPs were familiar with the *15-method* and had experience working with the method from the Danish feasibility study. This first group consisted of four HCPs (three GPs and one nurse) from two different practices, and they participated via video from their respective practices. The second focus group comprised four HCPs (two GPs and two nurses) who were from the same practice and had no prior experience with the *15-method*. This group was interviewed in person at their practice facility.

### Data storage

Data were stored on secure serves hosted by the Region of Southern Denmark at Odense Patient data Explorative Network (OPEN) [32] in compliance with the European General Data Protection Regulations.

### The interview guide

The interviews began with a brief introduction of the participants. This was followed by a general overview of the concept of SBI for alcohol problems in primary care and a brief background of the *15-method* to contextualize the study’s overall purpose of adjusting the *15-method* to Danish primary care.

Following the introduction, the patient interviews opened with a patient case on alcohol habits to offer the participants a scenario to reflect upon and talk from. The following questions were designed to help the participants reflect on the topic of alcohol and lifestyle in relation to primary care and on experiences using primary care as a setting for getting help to make a lifestyle change. After the initial general discussion on lifestyle and the role of primary care, the interview focused on topics such as experiences talking about alcohol with one’s GP or nurse, when and how they perceived it beneficial and meaningful to screen for potential alcohol problems, what the optimal SBI tool would look like in a primary care setting, and perceptions on what could facilitate or hinder a meaningful conversation on alcohol in a primary care setting.

For the HCPs familiar with the *15-method*, the discussion focused on different aspects related to the *15-method*, such as perceived strengths and limitations of the method, the method’s structure, potential target patient groups, and potential types of consultations in which the method could be utilized. Suggestions and ideas for e.g. visuals and the HCP manual were also included.

For the HCPs unfamiliar with the *15-method*, the interview focused more on the participants’ experiences with SBI for alcohol in primary care, with a strong emphasis on potential improvement areas. Topics ranged from financial aspects of the treatment options for alcohol-related problems in Danish healthcare, to details on specific workflows within a practice facility in which alcohol could be addressed more systematically.

The interviews were semi-structured [33], and each topic was initiated with open-ended questions to stimulate reflections and discussions. Closed-ended questions were used to provide details, clarify vagueness or ambiguities, and to test the interviewer’s understanding of statements or comments [33]. Prompts and examples were prepared for each topic to facilitate the discussion. The interview guide comprised three layers. The first layer consisted of overall research questions and hypothesized themes. The second layer consisted of specific research questions within each of the overall questions (e.g. details on barriers, facilitators, and the *15-method’s* material). The third layer comprised the interview questions and prompts based on the two prior layers. The interview guide had been discussed within the research group, and the interview questions had been subsequently read and commented upon by two lay persons unaffiliated with the study.

### Data analysis

#### Analytic framework

Analyses were conducted in an inductive-deductive process, also described as abductive analysis [34]. Thematic content analysis [35] was applied, in which statements, comments, and explicit opinions are grouped into themes on a semantic level [36]. The content analysis was guided by an initial codebook in a deductive approach with the three main topics (barriers, facilitators, and needs) as starting point, while potential new themes, insights, complex connections, and details were included during the analysis in an inductive approach [37, 38]. New themes and connections were then used to update the codebook and coding tree with details and nuances.

### Coding and analysis

Data were transcribed verbatim by a research assistant. PNS constructed the codebook and coded the transcripts. The codebook was informed by the interview guide and served as a start-list of codes that constituted the main branches in the coding tree, i.e. the three main topics investigated. Coding of transcripts was conducted in the same coding tree for both the healthcare professional interviews and the patient interviews with references to the data source. Themes were added as branches to the coding tree and were collapsed or renamed during the process as the level of detail increased. The themes were then visualized in a diagram to investigate overlaps and relationships between themes, and any new details were added to the coding tree. This approach helped identify themes from both participants who had experience using the *15-method* and those who did not for comparison. Finally, the identified themes were summarized in two focus areas that will form the basis for step two in the overall adjustment process of the *15-method*.

The analysis was conducted in iterative steps with repeated discussions among the research team members. Any disagreements were discussed until consensus and the research team discussed the progress between each step of the thematic analysis, i.e. familiarization with data, searching for themes, reviewing themes, and defining themes [35]. The information power [39, 40] in the present study was increased by: (i) the participants having a high level of knowledge on our specific research topic and its context, e.g., experiences with alcohol-problems, knowledge on the Danish primary care sector, and screening for alcohol-related problems, regardless of their knowledge of the *15-method* specifically, (ii) the narrow study research aim, (iii) the narrow theoretical framework applied, (iv) the strong dialogue with the participants via the interviewer’s knowledge of the intervention and setting, and finally (v) the analysis being conducted on selected cases with high level of detail and experience in the research topic in question (as opposed to more superficial exploratory cross-case analysis of larger groups) [40]. Analyses were conducted in Nvivo 12 [41] and did not include participant checking.

## Results

Within the three main topics investigated, we identified the following themes: Topic I, Barriers to addressing alcohol using the *15-method*, had two themes: (1) Not being on the same page, and (2) Structure and workflow. Topic II, Facilitators for addressing alcohol using the *15-method*, had three themes: (1) Interpersonal skills, (2) The patient’s perspective, and (3) Routines and interdisciplinary work. Topic III, Suggested adjustments and additions to the *15-method*, had no themes.

The themes were condensed in two focus areas for further work in the user workshops. The two focus areas were (1) Communication and material and (2) Integration to workflows.

**Fig. 1:**
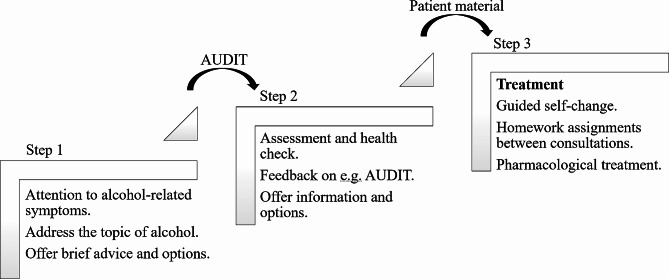
The three steps of the *15-method* Notes: Step 1 is embedded in already scheduled appointments and is not a stand-alone consultation. Step 2 can include blood sampling and clinical examination. Step 3 ranges from one to four consultations. The Alcohol Use Disorder Identification Test (AUDIT) and patient material can be given to the patient to complete between consultations (illustrated by arrows between the steps). Pharmacological treatment includes disulfiram, acamprosate, nalmefene, and naltrexone

**Fig. 2:**
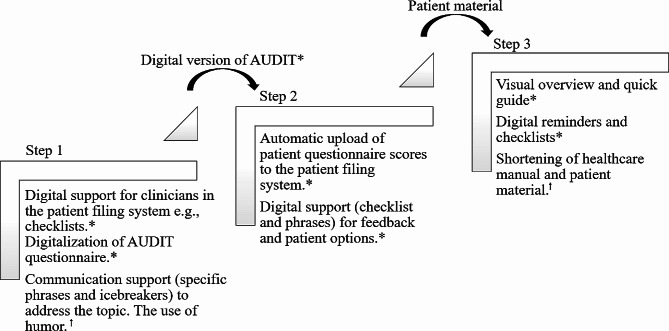
Suggested adjustments and additions to the *15-method* Notes: * refers to form, e.g. digitalization of specific tool like the Alcohol Use Disorder Identification Test. † refers to content, e.g. the tone of the written material

**Fig. 3:**
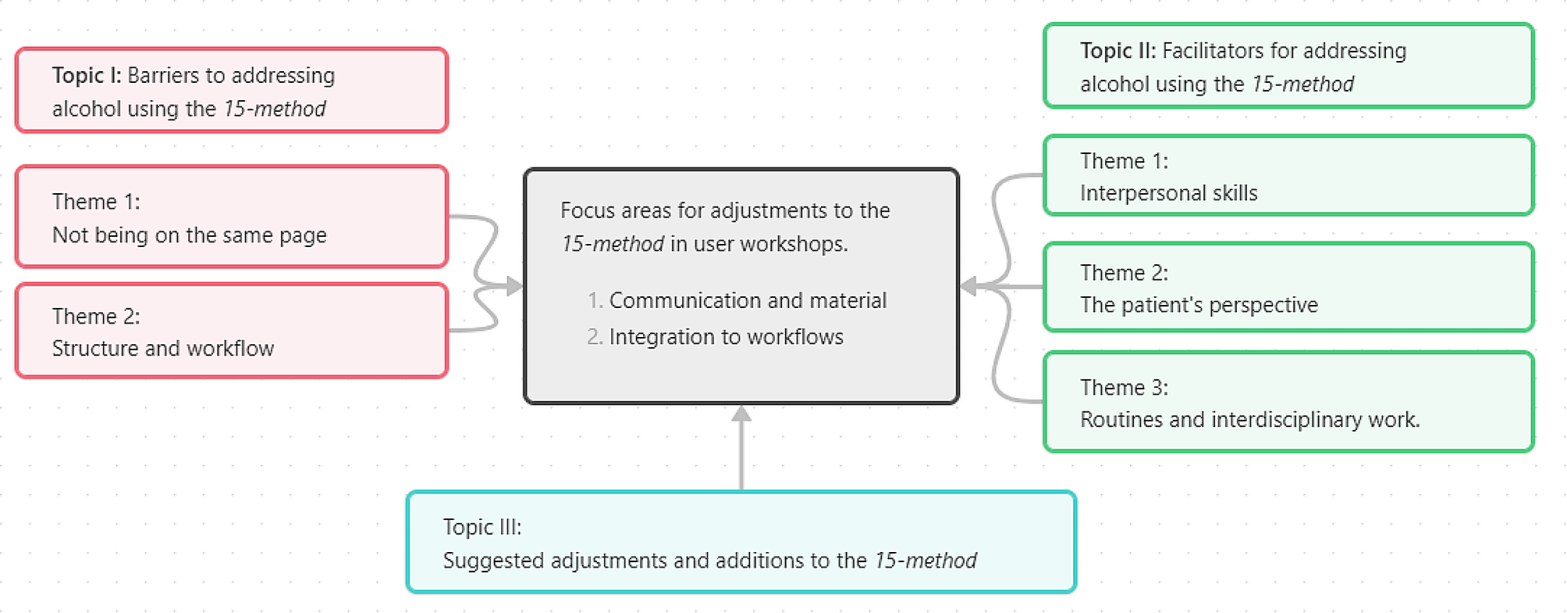
The relationships between identified themes of barriers and facilitators for addressing alcohol in relation to the *15-method* in Danish general practice Notes: Synthesis of themes into focus areas for user workshops. Interview data from four interviews with five patients and eight healthcare professionals

Figure 1 presents an overview of the three steps in the *15-method*. Figure 2 presents suggested adjustments and additions to the method according to each step. Finally, Fig. 3 presents the relationships between identified themes and focus areas.

There were no significant differences in data from the individual interview compared to data from the focus groups.

In the following section, parentheses indicate changes or explanations made by the authors.

### Topic I: barriers to addressing alcohol using the ***15-method***

#### Theme 1, not being on the same page

A common perception was that a consultation on alcohol habits is often skewed from the start, regardless of the use of the *15-method*. The approach to the topic of alcohol was considered to be paramount, but the main challenge is that the “right” approach is never the same. As the *15-method* is opportunistic in its approach, the HCPs considered its use was helpful for addressing alcohol habits in relation to a patient’s symptoms, e.g., what alcohol does to the body. As one patient said:

“…during the consultations, I realized that it (alcohol) actually affects many things, and I thought ‘well that makes a lot of sense’ because I had just thought my health issues were caused by something else - I had never made the connection that alcohol was causing me these issues. (Patient 2)

However, the symptom-based approach was also considered to risk pushing the patient away or being perceived as an insult if it was not attuned to the patient’s readiness to change. As one of the participating patients explained:

“If the doctor had said to me seven years ago: ‘Now listen: what’s bad for you, and what alcohol does to your body is…’ I would have said ‘yeah OK, whatever’ and wouldn’t have heard a thing”. (Patient 3)

One explanation to this opposing dynamic was the question of what was perceived as a problem. As some of the patients explained, the entire consultation is off-level if the HCP is trying to fix a problem that the patient does not see as a problem, but rather the solution to another problem:

“…you end up not talking about the problem but about the solution, and you can’t go any further down that road before you are so deep in trouble that everyone around you can see there’s a problem - and you eventually see it yourself.” (Patient 3).

The motivation to change and discuss alcohol was considered to be closely tied to the acceptance of one’s problems, but the courage to seek help could take months or years to build. As one patient explained:

“…I was afraid that it (alcohol) would become the explanation for every issue… I was afraid that I would have that label on me for good. Like ‘of course your stomach hurts, it’s because you drink too much’” (Patient 2).

Thus, the HCPs face a fine balance when they address a symptom with multiple plausible causes while keeping attuned to the patient’s readiness to discuss a potentially sensitive topic.

The HCPs recognized this difficult balance and pointed to the challenge of inquiring about health and lifestyle in a careful way. Some HCPs expressed difficulties in sensing the level of reflection and motivation regarding lifestyle and health when inquiring about alcohol and were concerned about pushing the patient away:

“It’s all about where the patient is… it can go both ways, whether one comes closer to the patient or pushes the patient away. If the patient at some point has had just tiny thought, then maybe it’s: ‘How great she just asked, now I can get help’, but if the patient is opposed to the thought, it can go the other way… it depends on where the patient is in all this” (HCP 2).

##### When to address a problem

Some of the patients perceived the HCP’s reluctance to discuss alcohol habits as another barrier:

“I briefly mentioned my thoughts on going into treatment for alcohol problems to my former doctor… she wanted no part of that conversation that’s for sure”. (Patient 4)

Overall, the HCPs did not have negative expectations when raising the topic of alcohol. Rather, they were more likely to hold back questions on alcohol out of a combination of structural constraints (presented below in theme 2) and respect for the patient’s agenda:

“… if you ask (about alcohol) and get an answer you must relate to, then it can’t really be a short conversation, can it? … it invites to something more and we must be constructive without pushing the patient away or making them feel we don’t take them seriously now that we were the ones asking… it has to have a trustworthiness that you want to help, and that’s why the setup has to be right.” (HCP 5).

#### Theme 2, structure and workflow

The workflow organization varies across general practices, and this was considered by some HCPs to explain some of the barriers to addressing alcohol problems. Practices varied in the extent to which the HCP (nurse or doctor) could address the topic of alcohol and in which type of consultation. From the patients’ perspective, these differences in continuity might be both a barrier and a facilitator. Building trust could for some take a long time and require a familiar face:

“…in the clinic where I go, I don’t have a feeling of anyone being ‘my doctor’… I feel like it’s back to square one every time I go there - I don’t even feel like they know my name… and I have contemplated asking for help for a long time, and boy have I tried to muster the courage many times… and then the feeling of not being seen – it knocks you right back, and it’s another three months or more before you even think of building the courage again.” (Patient 2).

The importance of building on a trustful relationship when inquiring about alcohol was shared by some HCPs:

“…Agree, alcohol habits are not the first thing we ask about.” (HCP 8).

“It seems too intruding (to ask about alcohol without any relation).” (HCP 6).

“The trust has to be there first.” (HCP 8).

In contrast to this view, some patients expressed how they might feel a higher degree of freedom in the consultation if they were talking to an HCP whom they did not know well.

Time constraints, which are a barrier to the use of SBI tools generally, were also found to be a barrier among HCPs, regardless of their knowledge on the *15-method*:

“…if you are behind schedule and think alright, maybe today is not the day we open up the gates.” (HCP 8).

“…there is a barrier here… the patients show up with a problem we have to manage, which makes it very problem-oriented, but we are under a lot of time pressure…” (HCP 5).

One aspect of finding time to screen for alcohol problems was related to the economic incentives. Although most of the HCPs stated that economic incentives would not change their behavior regarding screening for alcohol problems, a minority perceived these issues to be closely tied:

“…we are a sector driven by economic incentives… we do as we are told, if the money goes along with the job… and right now alcohol is just not part of that… so if we choose to have a conversation with a patient about alcohol, there is something else we can’t do with that patient.” (HCP 3).

The patients also recognized time constraints in general practice as a barrier:

“…we should treat people based on how ready they are and what type of problem they have… and treating them in a place where the time for treatment is available… and the tempo is lightning quick in general practice…” (Patient 4).

One patient expressed concern about the duration of consultations in general practice as a whole:

“For the patients who don’t see their doctor often I wonder if addressing alcohol is even possible, simply because the time isn’t there.” (Patient 5).

The other patients and all of the HCPs considered the duration of a consultation (usually 15 min in Denmark) sufficient to talk about alcohol but agreed that one or more follow-up consultations might often be necessary for further discussion of the situation.

### Topic II, facilitators for addressing alcohol using the ***15-method***

#### Theme 1, interpersonal skills

##### Connecting the dots

The symptom-based approach of the *15-method* was considered useful in connecting a symptom to the patient’s lifestyle. From the patients’ perspective, talking about lifestyle in relation to one’s health made sense:

“…asking about lifestyle challenges makes it easier to talk about the many factors that could be in play… focusing too intensely on one area can arouse resistance.” (Patient 5).

Most of the HCPs were aware of the balance in connecting the patient’s symptoms with potential lifestyle risk factors without arousing resistance in the patient:

“…a person comes to us with one or more symptoms. You then start to unveil the possible connection to alcohol… and then you move toward an acceptance of not some obscure disease, but alcohol, as the problem. And then you start working on the motivation towards making a change” (HCP 8).

#### Theme 2, the patient’s perspective

The most prominent facilitating factor for a conversation about alcohol was for the HCP to be able to see and understand the situation from the patient’s perspective. This ability was closely tied to interpersonal skills and attitudes among the HCPs, e.g. acceptance, empathy, and listening. This theme revolved around topics such as autonomy, goal setting, and readiness to change, but two aspects stood out: courage and risk factors vs. habit change.

##### Courage

All patients who had experience with alcohol-related problems agreed that building up courage to ask for help was a recurring theme in their experiences with alcohol. These patients also agreed that whether or not the HCP recognized the effort to build this courage could make or break the consultation:

“…this topic is so big, and we are all different… so it depends on where you are in life… are you talking to someone who has not considered treatment, or to someone who has signed up for treatment or to someone who has come out on the other side and now has to live sober… they all need completely different offers.” (Patient 3).

Regarding the structure of the *15-method*, it became clear that offering structure to cutting down or changing a habit was not always enough. For some patients, the most important catalyst for change was being recognized in their struggle and then to have a discussion on something bigger, e.g. what a life without alcohol could look like:

“…a question that keeps coming back is ‘sober, and then what?’ I mean, I could have sobered up, but what for? I needed someone who told me that there’s a life worth living being sober. And if you don’t have that answer, then trying to get sober ends up being a strange waste of time.” (Patient 3).

“…you need to accept these things and see your circumstances for what they are and then in some way experience a will to do something about it… what do you want the rest of your life to look like?” (Patient 4).

##### Risk factors vs. habit change

Changing a habit was viewed as a highly individual effort, but with strong social and cultural counterpressure. Several of the participants noted that it was not enough just to have the best intentions. Everyday life could make it difficult to change habits, as old habits, relationships, and peer pressure could interfere at any moment:

“…if you turn down a beer or a schnapps someone might look at you in a weird way. And that’s probably still true in a lot of settings. I think that part can be really hard to change… You need some serious health issues before you start going against the norm and say no.” (Patient 1).

“There’s a thin line between what is considered normal (to drink) and what is considered too much…” (Patient 2).

Patients and HCPs considered individualized follow-up consultations to be helpful in supporting the efforts to change habits. The consultations could be individually adjusted regarding the length of time between consultations and the patient’s homework assignments, e.g. identifying risk situations or making a plan for alternatives to drinking.

#### Theme 3, routines and interdisciplinary work

Several routines were suggested to help facilitate an increased focus on alcohol in general practice. One was to make alcohol a focus area in the practices’ regular reviews and quality improvement initiatives. A second was to make alcohol a part of existing focus areas such as hypertension or weight loss. A third was to make screening questions on alcohol habits a part of specific types of consultations such as yearly controls or newly diagnosed hypertension.

Interdisciplinary work was also a facilitating factor. As both nurses and GPs can use the *15-method*, the initial step of the method was considered to be an interdisciplinary effort. By identifying previously missed opportunities to inquire about alcohol habits, the HCPs could apply the opportunistic screening (step one of the *15-method*) during regular work routines. One such example was the use of “small talk” during blood sampling and electrocardiography (often done by nurses in the practice), which several HCPs agreed were opportune times to talk about lifestyle habits. These “small talk” opportunities utilized a further facilitating factor, which was to take advantage of prior established relations between patient and HCPs.

“…I have to make time for electrocardiography and blood sampling, and you can actually get around a lot of things during that time… I think it’s easier to ask about lifestyle habits when you are just talking to pass the time. It feels more natural.” (HCP 6).

### Topic III, suggested adjustments and additions to the ***15-method***

The suggestions for improvement related to the form and content of the method (see Fig. 2). The most requested feature regarding its form was a digitalization of the *15-method* to improve its flexibility and integration into the patient filing systems. A digitalized version was envisioned to fit into existing workflows, e.g. diagnostic procedures for hypertension that used predefined diagnostic algorithms in all practices.

“…we would have the questionnaire (AUDIT) in our patient records and could ask the patient to fill it in as part of the diagnostic process. Like with dizziness in elderly patients… or with all the other things we ask them to fill in like urinary input-output charts and depression scores and so on.” (HCP 8).

Other suggestions regarding the method’s form were, firstly, a visual referencing point that could help facilitate a discussion on alcohol, e.g. an illustration, a diagram, or an action card. The visual aid was perceived to help link health symptoms to the patient’s lifestyle and help continue the focus on alcohol into the next consultation. Secondly, posters or other visual material for e.g. the waiting room that could serve as icebreakers to the topic of alcohol or could help start a reflection among patients. Thirdly, a one-page overview of the method which could serve as a quick guide for easy referencing and structure. Suggestions related to the content of the method included the use of humor and lightening the tone in the written material.

### Focus areas for user workshops

The themes were condensed into two focus areas (Table 2). The focus areas will be addressed in future user workshops to finalize the adjusted Danish version of the *15-method*.

**Table 2:** Focus areas for adjustments to the *15-method* in user workshops The focus areas are based on a synthesis of four interviews with five patients and eight healthcare professionals (HCPs)

Focus area 1: Communication and material	Focus area 2: Integration to workflows
1. An emphasis on effective communicational skills in the material for HCPs including example phrases and sentences.	1. How to make screening for alcohol related symptoms a more integrated part of other screening procedures or diagnostics.
2. Increased help to HCPs and patients to connect lifestyle habits and symptoms. This includes a focus on how to present factual information without pushing the patient away and visual overview or illustrations.	2. Digitalization of the *15-method* including integration of questionnaire into patient filing systems.
3. The use of humor in the patient material.	3. Stronger emphasis on interdisciplinary workflows and use of routines and reminders.
4. Multiple approaches for the HCP to address the topic of alcohol.	4. Mix-and-match style treatment modules for higher flexibility.
5. Development of hand-out material and visual icebreakers which can serve as inspiration and as contact information.	

## Discussion

In this study, we investigated the perspectives of healthcare professionals and patients on the communication about alcohol problems in relation to the *15-method*, and how the method can affect barriers to addressing alcohol in Danish general practice. We further sought to identify aspects of the *15-method* that could be adjusted to increase its fit and effectiveness in Danish primary care.

The first topic, *Barriers to addressing alcohol using the 15-method*, underlined the difficulties experienced in approaching the topic of alcohol despite the willingness of both HCPs and patients to discuss the topic. Finding the appropriate approach and timing involved multiple aspects such as when to address a problem, the agenda within the consultation, and the organization of work in the practice. These aspects are well-known barriers to Screening and Brief Intervention (SBI) approaches for treatment of alcohol disorders [42].

The second topic, *Facilitators for addressing alcohol using the 15-method*, was much in line with previous research on the interpersonal skills and specific factors considered to facilitate habit change and to enhance patient outcome [43]. The *15-method* builds on Motivational Interviewing [44], which all the participating HCPs were familiar with and used to some extent, e.g. when asking permission before inquiring about lifestyle. The aspects that were mentioned by participants related to communication and interpersonal skills revolving around trust, genuineness, and empathy. These have been described previously as facilitating factors in a patient-client relationship [45].

Specific to the *15-method* structure, most of the participants found that a trustful relationship could be established in a single standard consultation, which in Danish primary care is fifteen minutes, regardless of the use of the *15-method*. This is important because the first step in the *15-method*, the opportunistic screening, has not been evaluated. Finn et al. [21] showed that the *15-method* had a non-inferior treatment effect on mild to moderate alcohol problems in primary care compared to specialist treatment in Sweden, but did not evaluate the initial step in the method. Screening frequency and treatment initiation rates for alcohol problems can increase with training and support [46, 47] and seem to increase with the resources allocated to these efforts [8], but sustaining these efforts with implementable solutions is notoriously difficult [6]. Two impeding factors are stigma [48, 49] and the complexity of the implementation context [47, 50]. The first step of the *15-method*, i.e. opportunistic screening, is embedded in scheduled appointments and is not a stand-alone consultation. This does not solve known SBI implementation issues such as time constraints and staff resources [51], but the method does offer differentiated treatment within the same setting as the screening, thus possibly addressing other known SBI barriers such as the uncertainty of HCPs in managing alcohol-related problems and raising the topic of alcohol in an appropriate context with adequate knowledge on effective treatment options [14, 52].

Training in the *15-method* is intended to increase the awareness of HCPs to potential alcohol-related issues and equip them with knowledge on how and when to address such problems. However, providing concrete next steps for treatment is not enough to ensure meaningful and high frequency screening. Theoretical knowledge can be difficult to apply [53], which is why we sought to identify which aspects of the *15-method*, especially in relation to communication and screening, were most important to adjust to maximize the method’s use and effectiveness in a Danish setting. Consistent with other studies on SBI for alcohol, a prominent challenge was how and when to address potential alcohol problems [14, 50, 54]. We found that if neither the HCP nor the patient approaches the topic of alcohol despite being willing to talk about it, the patient might get the impression that the HCP is trying to avoid the conversation because they do not care or do not have the time. Meanwhile, the HCP might hold back out of respect for the patient’s agenda or might think that the patient is not ready to discuss the topic or that there would be more time to discuss the topic in another consultation. Planned future work aims to address these challenges in relation to the *15-method*.

### Methodological considerations

The present study has several limitations. First, one patient knew one of the researchers, and some the HCPs knew of the researchers either through clinical work or through participation in the previous feasibility study of the *15-method*. Second, the participating patients were mostly male, and the sample size was relatively small. The results regarding the patients’ perspectives should be interpreted with caution, therefore, and cannot be extrapolated to a general patient population. Third, the participating HCPs had an interest in the research topic and could be considered early adopters or front runners in their field. Taken together, these points introduce the risk of bias, especially confirmation bias and availability bias [55].

The data were collected primarily through interviews performed via videoconference. Dialogue through such internet solutions can largely resemble face-to-face dialogue [56], but the online focus group interview requires an active moderator to avoid loss of intimacy and spontaneity due to the limited non-verbal communication [57, 58]. However, the online format also makes it easier to gather participants from different geographical locations and solves some logistical challenges while providing a safe environment for participants to discuss sensitive topics such as alcohol [58].

The participating patients had a wide range of experiences regarding alcohol habits and alcohol-related problems. These differences provided insights from different levels of alcohol-related situations as to how SBI procedures in Danish primary care could improve and how the *15-method* could be adjusted. Similarly, the HCPs who were unfamiliar with the *15-method* provided valuable visions and ideas for an optimal SBI tool. By combining these insights with experiences from HCPs familiar with the method, we were better able to identify useful adjustments to the *15-method*.

## Conclusion

Adjustments to the *15-method* may increase the method’s contextual fit to Danish general practice. Identified adjustments included greater focus on interpersonal factors, optimization of the method’s place in practice workflows, and adjustments to the method’s material. These adjustment areas will be addressed in user workshops to finalize a Danish version of the *15-method.*

### Electronic supplementary material

Below is the link to the electronic supplementary material.

Supplementary Material 1: Interview guides for the interviews with patients and healthcare professionals. Notes: The interview guide was structured in three layers. The first layer was the overall study research questions. The second layer was the specific research questions within each overall question. The third layer was the research questions re-framed as open- and closed-ended interview questions and prompts.

Supplementary Material 2: The Consolidated Criteria for Reporting Qualitative Research (COREQ) guideline checklist.

## Data Availability

The transcripts and coding tree are available in Danish upon request.

## Abbreviations

COREQ: Consolidated Criteria for Reporting Qualitative Research
GP: General practitioner
HCP: Healthcare professionals
MRC: Medical Research Council
OPEN: Odense Patient data Explorative Network
SBI: Screening and brief intervention
