# Supplementary material for: Adjusting the 15-method to Danish general practice: identification of barriers, facilitators, and user needs
Source: BMC Prim Care. 2024 Jul 6;25:248. doi: 10.1186/s12875-024-02508-z (PMC11227717; doi:10.1186/s12875-024-02508-z)
Supplement: Supplementary file 1 — Supplementary Material 1: Interview guides for the interviews with patients and healthcare professionals. Notes: The interview guide was structured in three layers. The first layer was the overall study research questions. The second layer was the specific research questions within each overall question. The third layer was the research questions re-framed as open- and closed-ended interview questions and prompts. [file 12875_2024_2508_MOESM1_ESM.docx]

Interview guide for the patient interviews

| **Layer 1.**  **Overall questions** | **Layer 2.**  **Research questions** | **Layer 3.**  **Interview questions** |
| --- | --- | --- |
| *Potential themes. Overall questions.* | *What do we as a research team want to know more about* | *Suggestions for specific questions, cases, prompts and follow-up questions* |
| What are some of the barriers to talk about alcohol from a patient perspective? | Do the participants have any positive or negative experiences with alcohol being addressed in a health care setting? - and if so, how?  Do the participants perceive general practice as a place to talk about alcohol habits - and why/why not?  Do the participants experience/fear/perceive the doctor-patient or nurse-patient relationship changes due to a conversation on alcohol?  How so? /why not? | Case example: a 50-year-old woman. She keeps a busy job. She enjoys wine after work and uses alcohol to “settle down” after a busy day. She drinks 3-4 glasses of wine each night to help fall asleep. She has reoccurring headaches and trouble getting a good night’s sleep. She was diagnosed with hypertension during a recent routine check-up.  What thoughts, if any, do you think the woman in the case has about her alcohol habits?  What would be an opportune place to discuss alcohol habits in general, when thinking about the Danish healthcare system?  Where would you look for information on alcohol or potential alcohol-related problems?  What do you think about talking to your GP or nurse about alcohol habits?  Who do you think can/cannot raise the question of alcohol in a health care setting?  Do you think your relationship to your doctor/nurse will change (or has changed) if you discuss alcohol?  In what way does the approach to the topic alter the conversation? |
| The perceived barriers to talk about alcohol with a healthcare professional | What type of barriers do the patient experience in their encounter with e.g. nurses, doctors, administrative staff in general practice?  What physical or concrete barriers have they experienced or perceive, e.g. time, location/consultation room, staff.  Do they experience any barriers related to communication?  How do the participant perceive barriers in relation to the 15-method in terms of: structure, approach, presentation of factual information, staff resources and interdisciplinary work? | How would you feel about talking about your alcohol habits with your practice nurse or your GP?  In what ways would it be difficult/easy - and why?  Do you find any differences between talking about alcohol with different staff groups?  Any situations that make it easier/harder? What happened?  In what way can you imagine alternative approaches? What would you find helpful in the conversation on alcohol habits (why/why not)?  How does logistics or availability of offers affect option for treatment? Have you any personal experiences or reflections you would like to share? |
| Perceived facilitators for addressing alcohol in general practice | Which elements/facilitators do the patient experience as helpful for a conversation about alcohol (problems)?  When does it feel transgressive/not transgressive to talk about alcohol with professionals in practice? | What would it take to make it easier to talk to others/the doctor about alcohol problems?  Is it easier/harder to talk about alcohol if the focus is on what it does to the body? / if it is brought up on the basis of a symptom / reasons for contact?  Do you feel that there may be a limit to what health professionals can/should ask about alcohol habits?  What, if anything, would be a limit for you in terms of talking about the topic (e.g. way of asking, time or the content of the question itself)? |
|  | According to the patients, how is alcohol consumption best/easiest discussed in a consultation?  Does the person in question wish to receive something (material, advice) at the first consultation?  To what extent does the patient want to take material home?  What increases the chances of the material being used?  What has worked well in previous instances? (e.g. elements, structure, ice-breakers) | How would you prefer a topic like alcohol is brought up – and when?  If you could decide, what would the best-case scenario for alcohol treatment in Denmark look like?  Who should be the first contact? How could the contact be made?  Would you like to receive any material? Should the material be online or physical?  Do you have any experiences with previous use of materials or procedures that have worked well? |

Interview guide for the healthcare professional interviews

| **Layer 1.**  **Overall questions** | **Layer 2.**  **Research questions** | **Layer 3.**  **Interview questions** |
| --- | --- | --- |
| Positive experiences from consultations regarding alcohol habits | Do they ask about alcohol? | Do you have experience talking to patients about alcohol?  Are you the ones bringing it up (staff group) How do you usually go about it?  Do you ever fail to ask? Why?  What could help you get better?  Have you experienced positive situations – a dialogue, a problem, a relationship, an opportunity or something else – that lead to a conversation on alcohol habits/change?  What happened?  Was it what you expected? Why/ why not?  Did you feel that your relationship with the patient changed by talking about alcohol habits? Why/why not? |
| Facilitators for talking alcohol in a consultation | What works for them?  How do they frame it and when? | In what situations do you ask about alcohol? Why?  What makes it easier?    Do you have a “go-to” sentence or way of framing the question(s)?  How much emphasis do you usually put on possible affects of alcohol on the patient’s symptom? |
| Experiences from consultations concerning other "difficult topics" | Difficult conversations and difficult clinical issues:  Which topics are perceived as difficult (if any)  How were these handled? Did it require structure, courage, knowledge, openness?  Did they miss anything in these situations that can be related to the subject of alcohol?  What were some aspects that worked well? | Can you see parallels to other difficult conversation topics? Is there something that repeats itself – such as structure, openness, courage, knowledge or something else you can draw on – or feel you need more of/try to be better at? |
| Barriers to addressing alcohol in a consultation | Is it a problem that they perceive as e.g. communicative, cultural, structural? Do they lack knowledge? | What can hold someone back from asking about alcohol habits?  What could help you to ask more often about patients' alcohol problems?  Is time a factor?  Experience?  Knowledge of alcohol? / treatment for alcohol problems?  Does the organization matter (e.g. workflow or who sees the patient first/at follow-up)? |
| Can we make the topic of alcohol easier to address? | Do staff feel they know when to ask?  Do they feel they know how to ask? | Is there anything you think will make it easier to talk about/ask questions/help you talk about the topic?  e.g. material?  Does it help to have a fixed structure?  Does accessibility matter? (online material, easy access to material)  Does the way you work in the clinic have an influence on the presentation/problem? How do you think it can be made easier to talk about/handle/follow up - based on how you work in the clinic? (e.g. follow-up, groups, screening, collaboration) |
| The staff's sense of when and how to inquire about alcohol | Do they feel that they know enough about alcohol (problems) and treatment to be able to help patients further? | As professionals, do you feel that you know when you can/should ask?  Is it easier if things are standardized? Or do you prefer a flexible approach?  Does it make a difference to you whether it is the doctor or the nurse who brings up the subject? Why?  What works well/less well? |
| Use of and need for material e.g. manual, hand-outs and patient material | Can they think of barriers to using material (e.g. manual or hand-outs)?  Do they perceive specific patient groups to be particularly positive (or negative) or more/less opportune in terms of hand-out material?  What would the perfect instruction manual for staff, and perfect patient material look like? | When does it feel right to use patient material and homework - and to what extent?  What makes you want to use it/not want to use it? Why?  Are there any consultations or issues where it is particularly relevant or good to have patient material ready? |
| What is essential for them to make an alcohol intervention work? | What gives the professionals more/less motivation to work with the topic of alcohol on a regular basis?  How do they see it most easily becoming a part of their daily work?  Examples of other projects/materials that have worked well/they still use/have found helpful. | In the ideal world, how can we help patients with alcohol problems? Material, options, place of contact, routines.  What would make it easier for you to work with this topic, in general?  What would that look like in your practice?  Do you have experiences from other projects that have work well? E.g. changes in routines, materials that work well. Favorite projects/initiatives that you still use? |
